# Physical therapy for end-stage hemophilic arthropathy: a case report

**DOI:** 10.1186/s12891-023-07056-8

**Published:** 2023-11-27

**Authors:** Zhen-zhen Gao, Hang Yang, Wen-bin Liu, Cui Xu, Shou-chang Xiang, Ling-cong Wang, Ya-jun Mao

**Affiliations:** 1 Department of Rehabilitation Medicine, The First Affiliated Hospital of Zhejiang Chinese Medical University (Zhejiang Provincial Hospital of Chinese Medicine), Hangzhou, Zhejiang 310006 China; 2 Department of Hematology, The First Affiliated Hospital of Zhejiang Chinese Medical University (Zhejiang Provincial Hospital of Chinese Medicine), Hangzhou, Zhejiang 310006 China

**Keywords:** Hemophilia, Hemophilic arthropathy, Physical therapy

## Abstract

This report introduces a young adult who has been in bed for more than ten years with end-stage hemophilic arthropathy. He didn’t have access to factor VIII (FVIII) in the early stage of hemophilia due to the high costs of clotting replacement therapy. As a result, he is experiencing some difficulties, such as joint contracture, muscular atrophy, severe pain, and poor function of cardiopulmonary. He came to visit us for a comprehensive rehabilitation program, and, finally, he achieved the basic goal of self-care in daily life.

## Key clinical message

Hemophilic arthropathy is a permanent joint disorder, particularly in hemophilia patients suffering from frequent and repetitive hemarthrosis. During the course of 20–40 years, more than half of the people diagnosed with hemophilia will eventually develop a severe form of it, with muscle atrophy, joint contracture, and limited functional activities. If conservative management fails, surgical options should be considered, including arthroscopic synovectomy, open synovectomy, and total joint arthroplasty. It puts a heavy burden on the family and society.

## Introduction

Hemophilia A is an X-linked recessive inherited bleeding disorder, caused by the missing or defective factor VIII (FVIII), with approximately 90% of repetitive bleeding occurring in the musculoskeletal system, and of these patients about 80% experience bleeding in the joint [1]. Eventually, bleeding events in this system develop severe hemophilic arthropathy, which is the result of repetitive intra-articular bleeding and synovial inflammation [2].

Hemophilic arthropathy is a permanent joint disorder, particularly in hemophilia patients suffering from frequent and repetitive hemarthrosis [3]. Between the second and fourth decades, many hemophiliacs develop severe articular destruction, with muscle atrophy, joint contracture, and limited functional activities [4, 5]. It puts a heavy burden on the family and society.

Hemophilic arthropathy plays an important role in significant crippling complications and disability that lead to the impairment of posture and balance, the restriction of joint motion, limitation of the activity of daily life (ADL), and prevention of social communication [6, 7]. There are four phases that a patient with hemophilic arthropathy goes through:

1. recurrent joint bleeding and acute pain;
2. synovial inflammation and joint remodelling;
3. muscle atrophy, joint contractures, and chronic pain;
4. severe limitation of functional activity and reduction of the quality of life.

Patients with end-stage hemophilic arthropathy are always required to get surgical arthrodesis or joint replacement therapy to improve the joint range of motion and achieve functional mobility [8].

This study reports on a 10-year bedridden young adult diagnosed with end-stage hemophilia arthropathy who achieved the goal of basic self-care in daily life through a comprehensive rehabilitation program instead of surgery. The study presents a nonsurgical method for the treatment and rehabilitation of patients with end-stage hemophilic arthropathy, which is also rarely reported in current literature. This is what our rehabilitation workers need to think about and explore.

## Case report

A 33-year-old young man was presented with joint contracture of his limbs for more than ten years. He was diagnosed with hemophilia A (severe type) due to his repetitive subcutaneous bruising at the age of eight months. With the severe deficiency of clotting FVIII, he experienced frequent spontaneous bleeding episodes in multiple joints and muscles, the oral cavity, and the subarachnoid. The left knee joint and the right elbow joint were more affected by hematoma. As the FVIII replacement therapy was unavailable to him for financial reasons. The painful experience of repeated bleeding and concerns about the safety of movement, the patient had been lying in bed and cared for by his mother since the age of 23, the reduction of daily activities and the lack of physical therapy resulted in gradual muscle atrophy, reduced joint movement, and the inability to walk and sit. Nowadays, it is easier for him to access the clotting FVIII. With the benefit of medical aid schemes, he would like to seek more treatment to see if he could be able to sit without support and have self-care abilities.

**Fig. 1:**
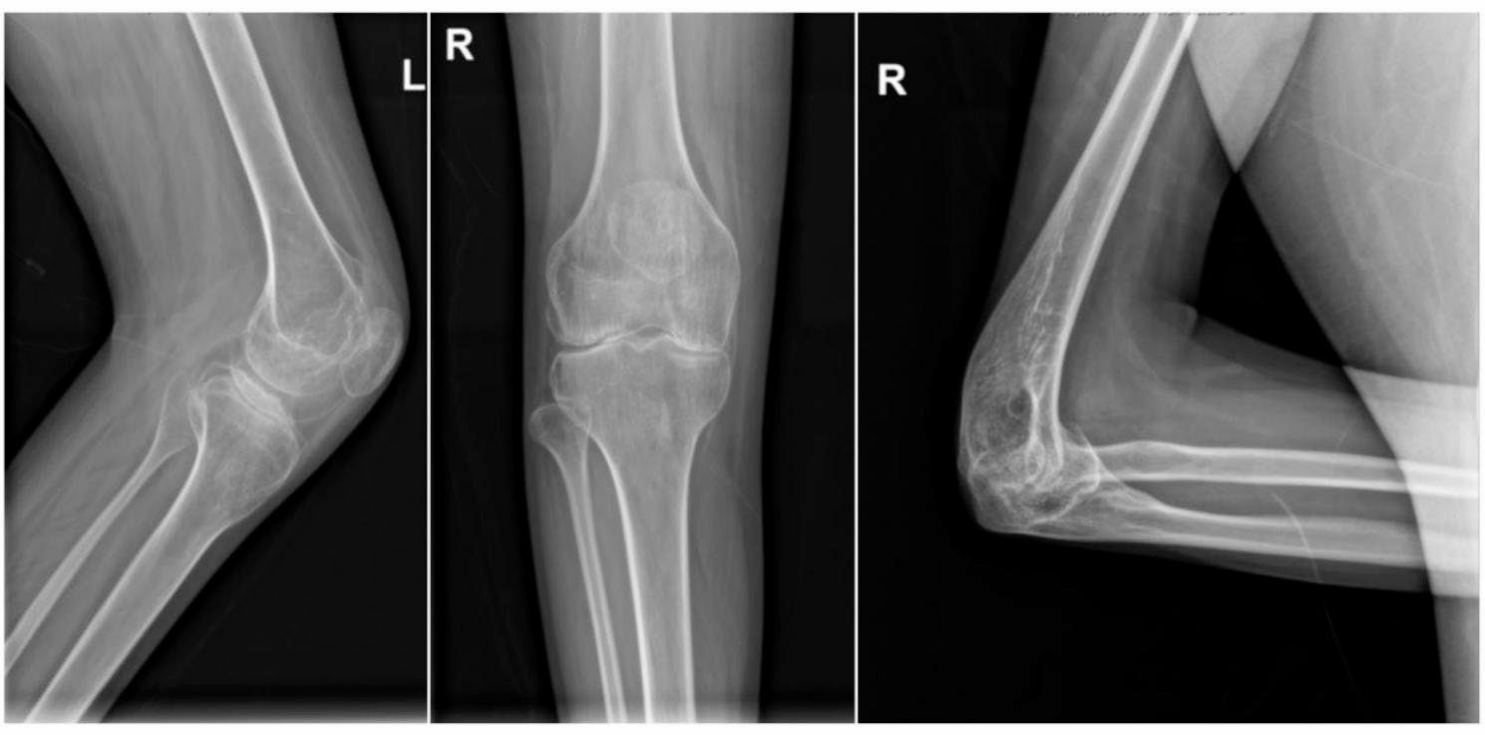
X-rays of knees and right elbow joints. Bone hyperplasia changes, loss of joint space, articular surface lack of smoothing

**Fig. 2:**
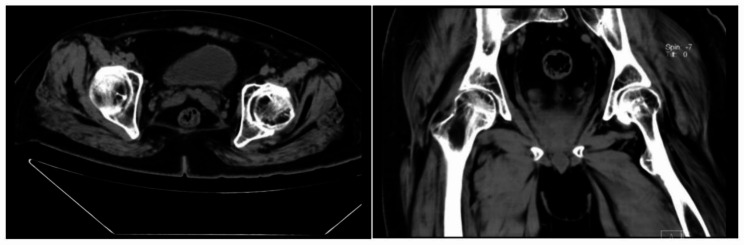
CT of the hip joint. Loss of the hip joint space, the uneven density of the femoral head, multiple cystic hypodense shadows under the articular surface, marginal sclerosis, sclerosis, and whitening of the articular surface bone

**Fig. 3:**
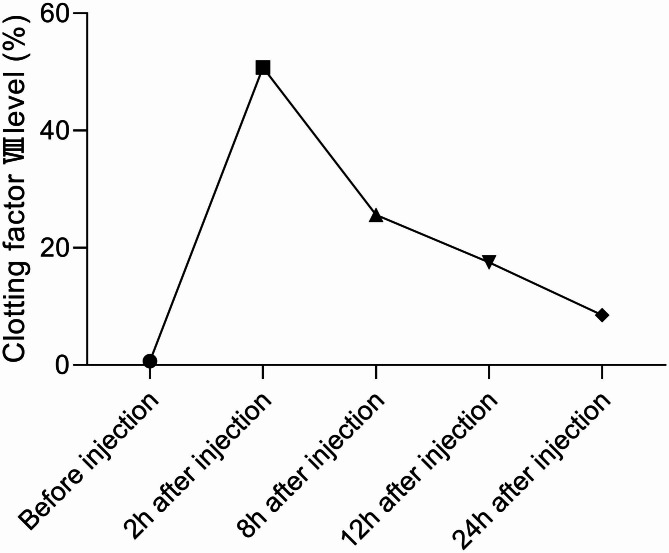
Clotting FVIII levels before and after injection

After completing the written consent form, the patient was evaluated using the radiograph evaluation of his large joints (Figs. 1 and 2), and the levels of his clotting factor (Fig. 3). Before and after eight weeks of physical therapy, he was given a detailed physical examination of his functional ability with a scoring system that includes the range of motion (ROM), visual analog scale (VAS) for pain, hemophilia joint health score (HJHS) [9], functional independence score in hemophilia (FISH) [10], and SF-36 quality of life score (SF-36) [11]. Adequate clotting FVIII replacement therapy is used to avoid hemarthrosis during physical therapy.

The interdisciplinary rehabilitation team consisted of a hematologist, an orthopaedic specialist, a rehabilitation physician, a physiotherapist, a nurse, and his mother. Considering his severe joint deformity, muscle atrophy, pain, and other complications, the orthopedic specialist recommended replacement surgeries for his hips (bilaterally) and left knee to extend his ROM of hips and knees to reach the goal of sitting up independently. However, both the patient and his mother refused it because of the risk of the surgery, and they wished to continue the conservative physical therapy, which was a challenge for the rehabilitation team. In terms of the family’s situation, the rehabilitation team tried to create a series of appropriate and progressive physical programs according to his condition.

The whole physical therapy program was under the guidance of the scheme of the amount of FVIII given by the hematologist to prevent intra-articular bleeding and other damage. The following was the replacement scheme: 2000 IU of the clotting FVIII (executive standard: JS20080050 manufacturer: Baxter AG) was injected intravenously 30 min in advance for every physical training [12].

Based on the careful physical assessment, our team planned an individualized and comprehensive physical therapy program (Table 1). Over the course of eight weeks of physical therapy, joint mobilization technique and muscle stretching technique were performed five times a week and muscle strength training was performed three times a week. From the third week, endurance training was conducted three times a week. The application of Rehabilitation engineering was carried out on the basis of the postural transfer technique and vestibular function training in weeks 4 to 6. Laser treatment and Chinese herbal fumigation were used to relieve patient pain when needed.

**Table 1:** Physical therapy program

Technique	Aim	Method	Training time	Frequency	duration
Joint mobilization technique	Maintain and upgrade ROM of the targeted joints	Active joint activity and passive joint activity for the major joints of the limbs, without obvious joint and muscle pain.	Each targeted joint should be moved 10 times in all directions, for a total of 20 min per time.	5 times / week	Throughout the course. (from 1–8 weeks)
Muscle stretching technique	Relax the contracture of the muscle and ligament; upgrade mobility of joint	Passively stretch the contracted and tense muscles around the major joints of the limbs in the opposite direction, with a painless or mild pain range	Each joint should be stretched for 10s in all directions, rested for 20s, repeating 10 times for a total of 60 min.	5 times / week	Throughout the course. (from 1–8 weeks)
Muscle strength training	Improve the muscle strength	Progressive resistance training with elastic bands for the main muscle groups of the limbs; bridge exercises for the trunk core muscle groups; it was under the principle of no obvious muscle soreness the next day.	Choose 3 to 5 major muscle groups each time, 10 times/set for each muscle group, repeat 2 times for a total of 20 min.	3 times / week	Throughout the course. (from 1–8 weeks)
Endurance training	Develop the endurance of exercise	Aerobic exercise training for limbs in bed, continuous sitting training, with RPE being 11–13 points and no obvious fatigue the next day	A total of 30–40 min	3 times / week	Form 3–8 week
Postural transfer technique	Improve the ability of daily life	Rollover training in bed; planar transfer training; bed-wheelchair transfer training	A total of 10–20 min	Twice a day.	From 4–6 week
Vestibular function training	Relieve the dizziness	Progressive sit-up exercises; vestibulo-ocular reflex exercises; dynamic drive exercises	A total of 30 min	Once a day	From 4–6 week.
Rehabilitation engineering (Fig. 4)	Help transfer and improve the ability of life.	The operation of the integrated electric bed-wheelchair device, including driving, steering, and safety	A total of 20 min	Once a day	From 6–8 week
Laser treatment	Alleviate pain	Irradiating the painful area with He-Ne laser; the power is 5-10mW, and distance is 30-80 cm.	A total of 5–10 min	Once a day; no more than 15 times for the same area.	When necessary.
Chinese herbal fumigation	Alleviate pain	Boil some selected Chinese herbs with Chinese medicine fumigation instrument, then aimed at the painful part with the distance at 30 ~ 40 cm.	A total of 20 min	Once a day.	When necessary.

The comparison of the rehabilitation assessments before and after two months of physical therapy (Tables 2 and 3). Before physical therapy, the patient’s main joints in the limbs are all subject to varying degrees of limitation. The range of flexion of the right elbow is between 55° and 77°, the range of flexion of both hips is less than 40°, the range of flexion of the left knee is between 50° and 80°, and the range of flexion of the right knee is between 5° and 30°, which limits the patient’s activities such as dressing, eating, sitting up, standing, and walking. In addition, the patient also has joint pain issues. In terms of specialized evaluations related to hemophilia, his Hemophilia Joint Health Score is 51 points, and the Functional Independence Score in Hemophilia is 8 points. The score of SF-36 also indicates a serious decline in the patient’s quality of life. After physical therapy, the patient’s main joint range of motion has improved. The flexion range of the left shoulder joint increased by 20°, the range of motion of the right elbow joint increased by 13°, the flexion range of the left hip and right hip increased by 25° and 34°, respectively, and the flexion range of the left knee and right knee increased by 18° and 11°, respectively. The patient’s HJHS decreased by 9 points, benefiting from improvements in range of motion, pain, and muscle atrophy. With the electric bed-wheelchair device, patients can transfer between the bed and the electric wheelchair, and control the electric wheelchair to go out, which improves the patient’s functional independence and quality of life.

**Table 2:** Range of motion in the major joints of the limbs

joint		movement		Before	After
shoulder	Left		flexion	0°~140°	0°~160°
			extension	0°~38°	0°~40°
	right		flexion	0°~120°	0°~125°
			extension	0°~35°	0°~40°
elbow	Left		flexion	0°~135°	0°~140°
	right			55°~77°	50°~85°
hip	Left		flexion	0°~40°	0°~65°
			extension	0°~5°	0°~10°
	right		flexion	0°~24°	0°~58°
			extension	0°~20°	0°~20°
knee	Left		flexion	50°~80°	45°~93°
	right			5°~30°	5°~41°
ankle	Left		flexion	0°~5°	0°~5°
			extension	0°~5°	0°~7°
	right		flexion	0°~5°	0°~7°
			extension	0°~10°	0°~13°

**Table 3:** Pain, joint function, mobility, and quality of life scores

Assessment content	Before	After
VAS	3	1
HJHS	51	42
FISH	8	11
SF-36	70.7	98.15

VAS: Visual Analogic Scale for Pain; HJHS : Hemophilia Joint Health Score; FISH: Functional Independence Score in Hemophilia; SF-36: the MOS item short-form health survey

## Discussion

Intra-articular bleeding (hemarthrosis) is the most common musculoskeletal problem in patients with hemophilia, running into a vicious cycle; hemarthrosis-synovitis-hemarthrosis, that makes the condition become a chronic and permanent joint disorder [13]. The pathomechanism of hemophilic arthropathy is complex and poorly understood, which involves hypertrophic synovitis, osteopenia, and damage to the cartilage and subchondral bone [14]. The pathomechanism in hemophilia often occurs simultaneously and interacts with each other [15] when a patient is at the age of learning to walk and older, generally for the same joints which are commonly involved in large joints; namely in the knee, elbow, ankle, hip, and shoulder [4]. Inflammation is also involved in this progressive pathomechanism, associated with iron, inflammatory cytokines, neo-angiogenesis, and recurrent released hemoglobin [8]. As such, the incidence of hemophilic arthropathy is relatively high in young patients and easily progresses to end-stage arthropathy if not well managed [16].

Surgical treatment is necessary for hemophilia patients with acute bleeding, subacute bleeding, chronic hemophilic arthropathy, or hemophilic pseudotumor and related fractures [17]. During the course of 20–40 years, more than half of patients will develop a severe arthropathy with functional disability. If conservative management fails, surgical options should be considered, including arthroscopic synovectomy, open synovectomy, and total joint arthroplasty [1]. It is also believed that an aggressive approach needs to be adopted for the young adult who deserves optimal physical health while his musculature is still in good condition. For end-stage hemophilic arthropathy patients, total joint replacement is the optimal choice to relieve pain, correct deformity, and improve functional activities [18].

In this case report, we presented a young patient whose large joints are in stage V (end-stage) based on radiographic evaluation and the Arnold-Hilgartner classification scale [19]. He was recommended for surgery (total joint replacement of hips and knees) to reconstruct his joints in order to be fully rehabilitated. Although there may be some risks of infection, bleeding and revision in hemophilic arthropathy surgery [20, 21], joint arthroplasty is still an important means to improve joint function with low complications through good surgical techniques and hematological management [22, 23]. However, this patient required multiple joint replacement surgery. After careful consideration of all potential option of risks and benefits, the patient chose conservative treatment. In terms of the severity of his degree of joint destruction and the irreversible progression of his disease, he may be faced with worsening muscle atrophy, joint damage, bone fracture, recurrent pain, and the failure to be fully rehabilitated. Furthermore, he may be faced with more risks of surgery in the future. As a result, it is a challenge to make an individualized and appropriate rehabilitation program for him.

It is believed that the first line of treatment for hemophilic arthropathy is physiotherapy with a factor replacement regimen. However, experts still think when all strategies for the treatment of joint disease fail, joint related surgeries may need to be performed [18]. Scientists have turned to non-surgical methods for the treatment and rehabilitation of hemarthrosis patients [24]. Physical therapy is essential for reducing disability and improving the quality of life [25]. Individualized rehabilitation programs could have a positive impact on the management of pain, joint health, and disability improvement [26]. Joint mobilization and muscle stretching techniques have been reported as effective methods for improving joint health, range of motion and joint pain [27–30]. This is consistent with what we observed in this patient.

Currently, patients with end-stage hemophiliac joint disease often require surgery to improve their function [31]. For this patient, the long-term bed living, muscle atrophy, and poor cardiopulmonary function were challenges that the team should consider to balance the risks and benefits of the surgery. Fortunately, the effect of physiotherapy is encouraging, which also indicates that an appropriate rehabilitation program should be part of the surgical treatment of end-stage hemophiliac arthropathy.

**Fig. 4:**
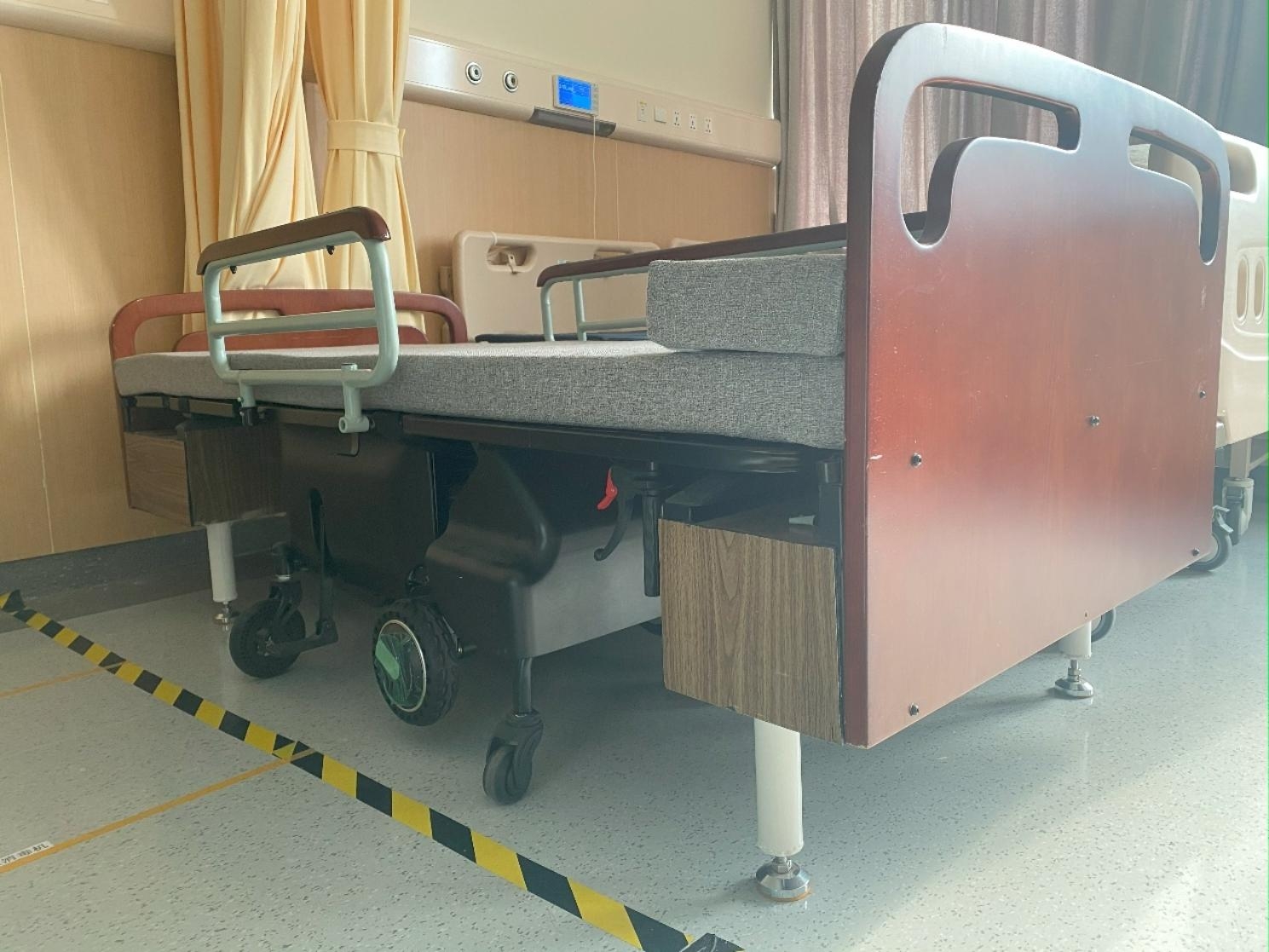
Bed and electric wheelchair integrated equipment

However, physical therapy still has some limitations. In this case, it is almost impossible for him to achieve walking, even though the ROM of his stiff joints improved. Therefore, with the help of rehabilitation engineering, he is equipped with an integrated electric bed-wheelchair transfer device (Fig. 4) to help him go outside. His choice to use this auxiliary device highlighted his desire to mobilize and improve his ability to take care of himself. While he was transferred to this electric bed-wheelchair, he suffered severe vertigo several times, which was unexpected and rarely mentioned in current reports of hemophilia patients. In hemophiliac arthropathy, the destruction of cartilage, synovial membrane, and ligament structures changes the information of joint mechanoreceptors, resulting in decreased flexibility and proprioception [32, 33]. At the same time, prolonged supine position may lead to vestibular dysfunction. This raised another challenge: how to improve the performance of static and dynamic adaptability of patients through proprioception and vestibular function rehabilitation.

## Conclusions

For patients with end-stage hemophilia arthropathy, surgery is not the only option to improve mobility. An individualized and comprehensive rehabilitation program can also play an important role in improving joint ROM, enhancing muscle strength and endurance, relieving pain, and improving activities of daily life and quality of life.

## Data Availability

The datasets are available from the corresponding authors on reasonable request.
